# *In vitro* biomechanical study of a novel fixation device for lumbar spondylolysis

**DOI:** 10.3389/fbioe.2025.1669734

**Published:** 2025-12-08

**Authors:** Jie Yang, Lijun Wang, Zecheng Cai, Wanzhong Yang, Rudong Chen, Rong Ma, Zhaohui Ge

**Affiliations:** 1 Department of Orthopedic, General Hospital of Ningxia Medical University, Yinchuan, China; 2 The First Clinical Medical College, Ningxia Medical University, Yinchuan, China; 3 Shandong Weigao Orthopaedic Device Co., Ltd., Weihai, Shandong, China

**Keywords:** lumbar spondylolysis, internal fixation system, in vitro biomechanics, pedicle screw, novel pedicle screw-rod-domino-rope fixation device

## Abstract

**Background:**

Lumbar interbody fusion effectively treats isthmic spondylolysis but causes motion loss, adjacent segment degeneration, and potential neurological complications, limiting its use in adolescents. While intrasegmental fusion offers a promising alternative, current fixation devices remain experimental with notable limitations. This study uses *in vitro* biomechanical methods to evaluate the biomechanical performance of a novel lumbar spondylolysis fixation device.

**Methods:**

Seven fresh calf lumbar specimens were used in in vitro biomechanical experiments to establish intact lumbar models (INT), lumbar isthmic spondylolysis models (IS), pedicle screw hook models (PSH), pedicle screw U-shaped rod models (PSU), and a novel pedicle screw-rod-domino-rope fixation device (PSDR). Each specimen underwent a pure torque of 7.5 Nm, and the range of motion (ROM), lumbar spinal stress (LSS), and device attachment stress (DAS) were measured under flexion-extension, lateral bending, and axial rotation.

**Results:**

There was no significant difference in the ROM values among PSH, PSU, and PSDR (P > 0.05), and there was no significant difference compared to INT (P > 0.05). In the flexion-extension state, the PSU exhibited the largest ROM. Apart from the flexion-extension state, PSDR’s ROM is numerically greater than that of PSH and PSU. PSDR had the highest LSS value among the three device groups, which was significantly greater than those of PSH and PSU (P < 0.05). PSDR had the lowest DAS value among the three device groups, which was significantly lower than those of PSH and PSU in the flexion-extension state (P < 0.05).

**Conclusion:**

PSDR can effectively increase the stress at the spondylolysis site and reduce the stress at the device attachment point in lumbar spondylolysis repair, outperforming PSH and PSU.

## 1 Introduction

Lumbar spondylolysis is a condition caused by chronic, repetitive stress acting on the lumbar spine, characterized by unilateral or bilateral bone discontinuity or defects in the transitional area between the superior and inferior articular processes and transverse processes, commonly observed in the L5 vertebral body (Chung and Shimer, 2021; Mohile et al., 2022). Lumbar spondylolysis is a major cause of low back pain in adolescents, with a common onset age of 15 years (Choi et al., 2022). Early lumbar spondylolysis tended to be treated conservatively for 6 months. For patients whose symptoms remain unrelieved after conservative treatment, surgical intervention should be considered (Tanveer et al., 2021). Currently, the most commonly used repair method is intrasegmental fusion, which has given rise to a series of classic surgical techniques since its introduction (Buck, 1970; Morscher et al., 1984; Zahid et al., 2003). However, owing to the high incidence of complications, pedicle screw fixation methods have been gradually replaced. Although pedicle screw-pedicle-hook fixation (PSH) (Tokuhashi and Matsuzaki, 1996) and pedicle screw-U-shaped rod fixation (PSU) (Altaf et al., 2011) have addressed some of the shortcomings of traditional surgical techniques such as rigid fixation, they still pose a risk of surgical failure due to excessive stress, which can lead to fractures at the attachment sites (spinous process roots, laminae).

**FIGURE 1:**
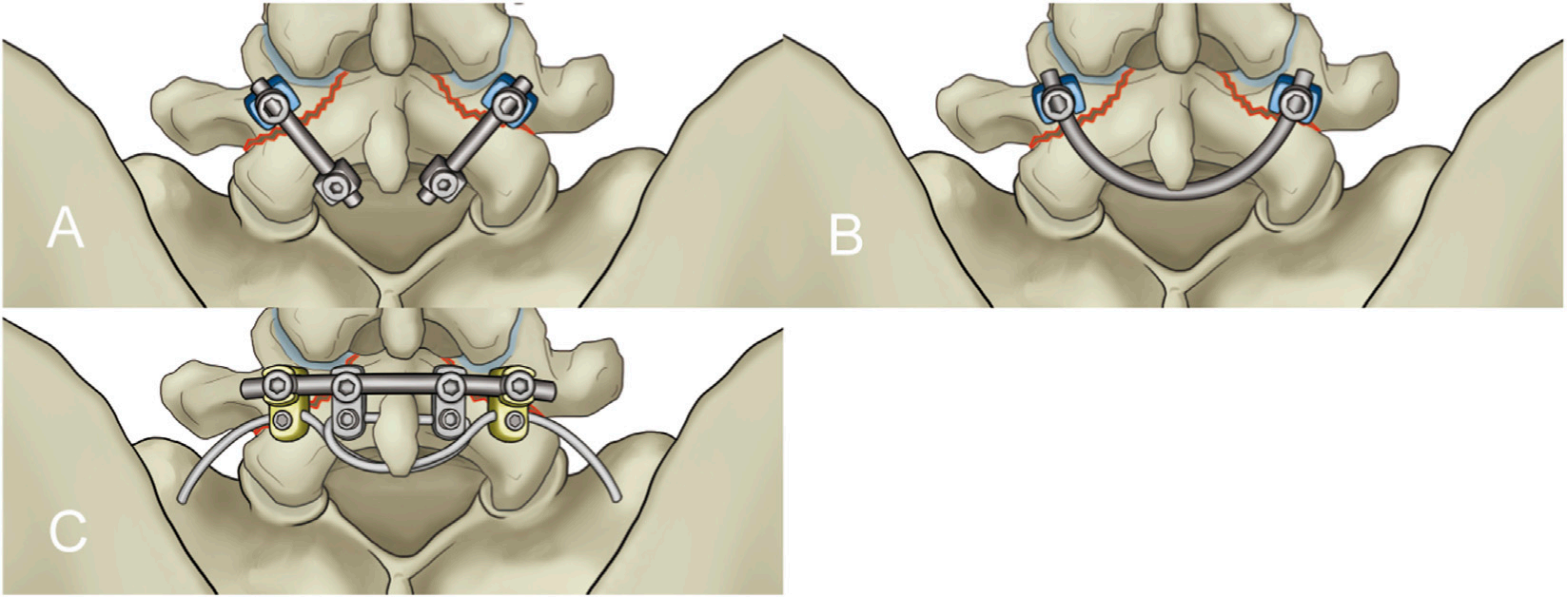
Schematic diagrams of the three fixation devices: **(A)** Pedicle screw-hook fixation **(B)** Pedicle screw-U-shaped rod fixation **(C)** Pedicle screw-rod-domino-rope fixation.

According to Wolff’s Law (Frost, 1994), applying appropriate stress stimulation to the fracture ends enhances bone healing. This study designed a novel lumbar spondylolysis fixation device, the pedicle screw-rod-domino-rope fixation device (PSDR) (Figure 1) (China Patent Number: ZL202120152603.9). Our preliminary application of three-dimensional finite element analysis (Zhou et al., 2023) showed that PSDR optimizes the stress distribution at the implant-bone interface while maintaining biomechanical stability similar to rigid fixation, effectively promoting bone healing in spondylolysis. This study aimed to explore the potential clinical application value of PSDR by comparing it with PSH and PSU using *in vitro* biomechanical methods, thereby providing a biomechanical reference for its clinical application.

## 2 Materials and methods

### 2.1 Specimens

This study was approved by the Ethics Committee of the General Hospital of Ningxia Medical University (KYLL-2022–1,137). The specimens studied were obtained from a slaughterhouse in Yinchuan, China, including seven fresh lumbar spines from calves aged approximately 6 months. When selecting the specimens, radiographic examination was performed to exclude fractures or lumbar deformities. CT was used to measure the bone mass of each specimen, excluding those with abnormal bone mass.

The muscles and soft tissues surrounding the vertebral bodies were removed according to the anatomical structures. In contrast, the vertebral bodies, intervertebral discs, endplates and ligaments were retained to form a functional spinal unit for testing. L2 and L6 were embedded in polymethylmethacrylate, and all specimens were stored in double-sealed bags at −30 °C when not in use. Prior to the experiment, each specimen was gradually heated to room temperature (20 °C ± 3 °C) until it was completely thawed. Specimens were wrapped in gauze soaked in saline to prevent dehydration.

### 2.2 Establishment of surgical model

**FIGURE 2:**
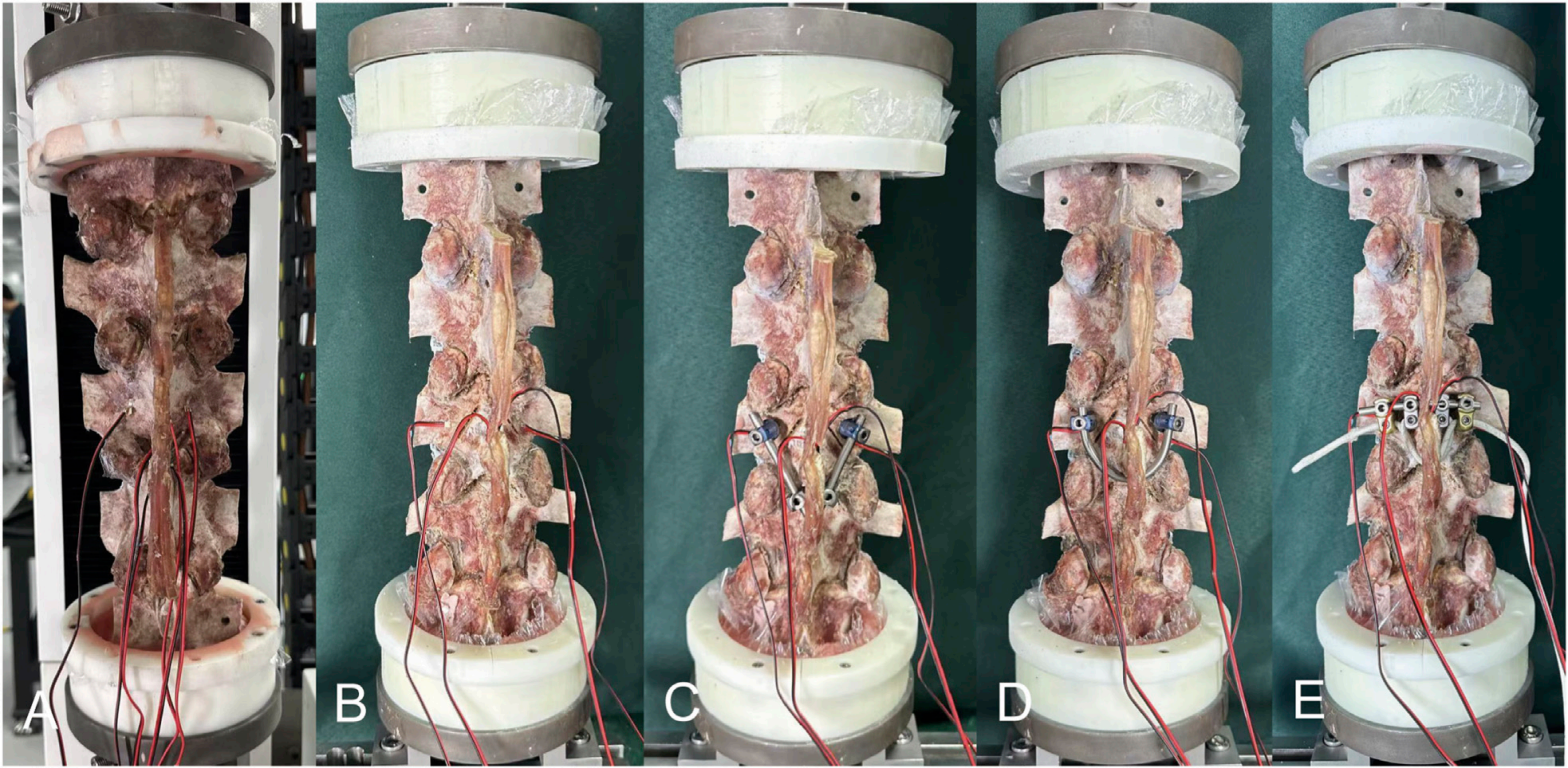
The five fixation devices used in the experiment: **(A)** The intact lumbar models, **(B)** The isthmic spondylolysis models, **(C)** The pedicle screw-hook fixation, **(D)** The pedicle screw-U-shaped rod fixation, **(E)** The pedicle screw-rod-domino-rope fixation.

**FIGURE 3:**
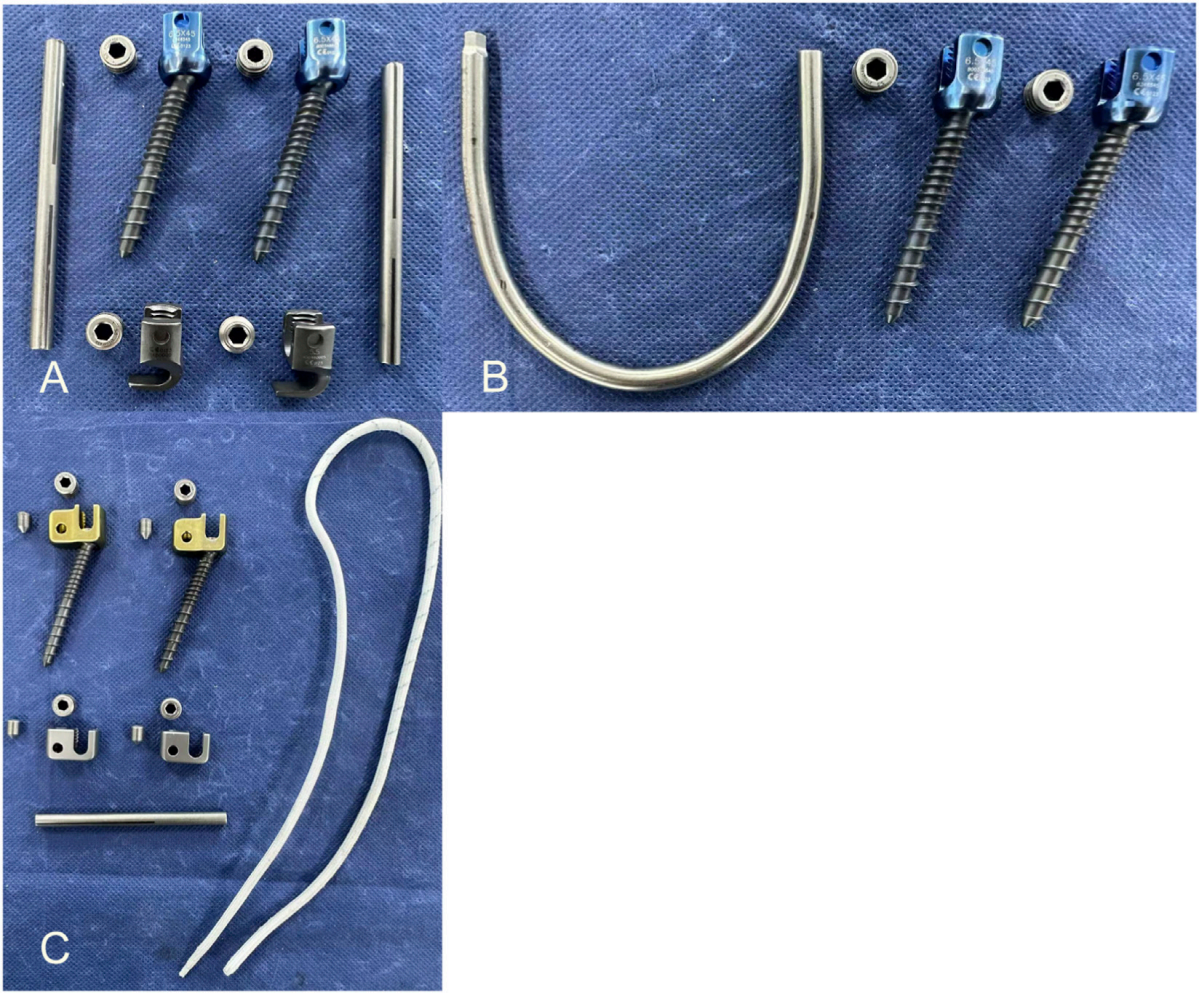
The implants used in the experiment: **(A)** The implants were used in pedicle screw-hook fixation device implant **(B)** The implants were used in pedicle screw-U-shaped rod fixation device implant **(C)** The implants were used in pedicle screw-rod-domino-rope fixation device implant.

**FIGURE 4:**
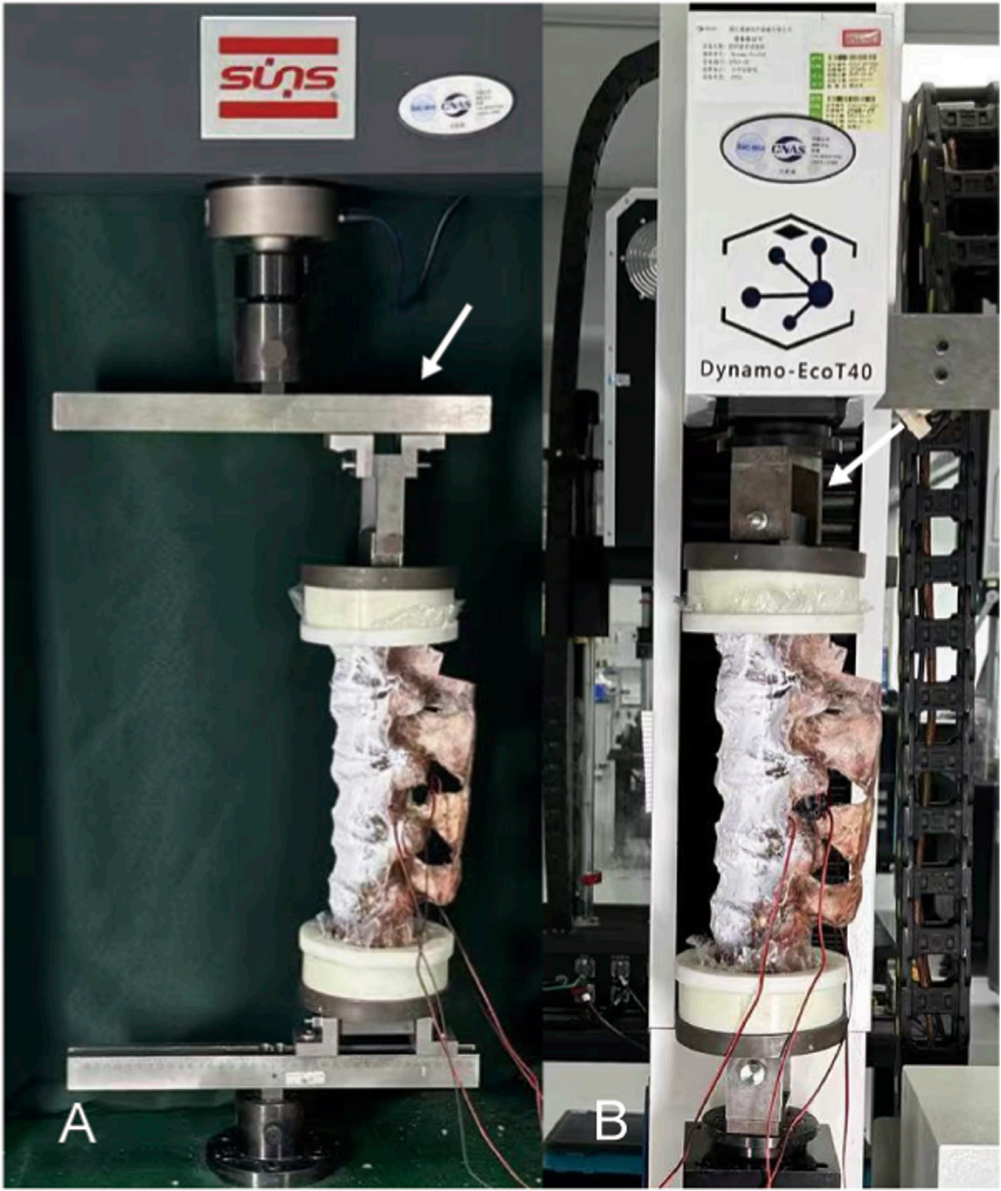
The mechanical testing machine used in the experiment: **(A)** Torsion test machine can be produced a torque of 7.5 Nm on the cephalic side of specimen (white arrow). **(B)** After the special fixture is fixed with the universal testing machine, it can provide a pure torque of 7.5 Nm on the cephalic side of specimen to produce flexion, extension, and lateral bending motion (white arrow).

**FIGURE 5:**
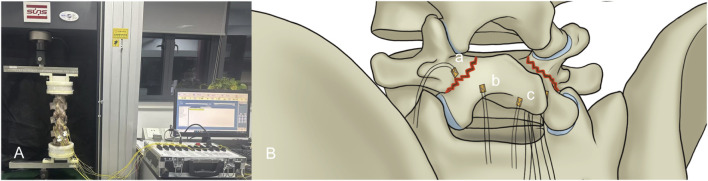
Stress measurement methods: **(A)** The strain gauge collects strain data measured by the strain gauge, substitutes it into the elastic modulus of the lumbar vertebrae, and calculates the corresponding stress value. **(B)** Strain gauge placement during the experiment and corresponding measured strains: **(a)** Lumbar spondylolysis stress **(b)** Pedicle screw-hook fixation device attachment stress **(c)** Pedicle screw-U-shaped rod device and pedicle screw-rod-domino-rope device attachment point stress.

Each specimen was measured in the following five groups: (1) intact lumbar (INT, Figure 2A), (2) isthmic spondylolysis (IS, Figure 2B), (3) pedicle screw-hook fixation (PSH, Figure 2C), (4) pedicle screw-U-shaped rod fixation (PSU, Figure 2D), and (5) pedicle screw-rod-domino-rope fixation (PSDR, Figure 2E). All the models were prepared by an experienced spine surgeon.

Before the experiment, seven specimens were subjected to flexion-extension, lateral bending, and axial rotation movements in their intact state to obtain the range of motion (ROM) for each movement mode. A multifunctional high-speed oscillating saw cutter (Shizhong Electric Appliances, China) was used to create a 2.0 mm defect in the bilateral isthmic regions of L4, simulating the creation of a spondylolysis model at the L4 vertebral body. Subsequently, different surgeries were performed in sequence, using the lumbar pedicle screw fixation system (Weigao Orthopedic Materials Co., Ltd., China) as the surgical instrument: pedicle screws (screw cap diameter 6.5mm; screw length 45 mm), lamina hooks (5.5 mm), PSDR pedicle screws (screw cap diameter 6.5mm; screw length 45mm; cable hole diameter 4 mm), PSDR domino device (cable hole diameter 4mm; rod hole diameter 5.5 mm), rods (5.5 mm), and polyethylene terephthalate (PET) cable device (diameter 4 mm) (Zimmer Biomet Holdings Inc., America). Before fixing the device, each set of specimens was extended posterior. Once the device was installed, it was restored to its normal state, simulating the intraoperative compression of the lumbar isthmus to ensure its stability and integrity (Figures 3A–C).

1. PSH: After removing a portion of the lamina at the inferior margin of both laminae of the vertebral plates, a hook applicator was used to insert the lamina hook, and a rod was connected to the pedicle screws on both sides. Finally, a set screw was used to secure the screw (Figure 1A).
2. PSU: The transverse rod was bent to a suitable angle using a bending device; the U-shaped rod was then looped around the root of the L4 spinous process and connected to the pedicle screws on both sides. Finally, the construct was secured with a set screw (Figure 1B).
3. PSDR: Specially made pedicle screws were placed, part of the spinous process was removed, the transverse rod was connected across the spinous process to the pedicle screws on both sides, a domino was fixed to each side of the transverse rod adjacent to the spinous process, and the transverse rod was secured with a set screw. Finally, the rope device was passed through the pedicle screw and the domino on one side, bypassed the root of the spinous process, and was connected to the device on the opposite side. The rope device was then tightened with a rope tensioner and fixed with a set screw. During implantation, a pre-tightening force of 200 N was applied to the tensioned rope, and the rope device was secured with a top wire (Figure 1C).

### 2.3 Biomechanical testing and protocol

The equipment used in this study was sourced from the DECANS Medical Biomechanics Experimental Center (DECANS Medical Device Co. Ltd., Zhejiang, China). An electronic universal testing machine (SUNS, Shenzhen, China) was used to generate the flexion-extension and lateral bending of the test specimens (Figure 4A), whereas a four-column tensile/torsional testing machine (Dynamo-EcoT40, Germany) was used to produce the axial rotation of the specimens (Figure 4B). With the assistance of specific fixtures, these two machines could provide a pure torque of 7.5 Nm (with a loading speed of 1°/s) to the head of the sample, enabling it to generate motion with six degrees of freedom, including flexion/extension, left bending/right bending, and left rotation/right rotation. A 7.5 Nm bending moment was generated via an eccentric vertical load, inherently producing axial force. Eccentricity was unfixed to simulate posture-dependent dynamics. Despite axial-load influence on ROM and stress, all devices experienced comparable conditions under a constant bending moment, indicating that differences mainly reflect device mechanics.

Based on the standardized testing recommendations for spinal implants and methods reported in the relevant literature, no axial preload was applied to the experimental materials, indicating that the influence of simulated self-gravity on lumbar range of motion (ROM) and spinal stress was not considered (Cai et al., 2022; Panjabi, 1988). Based on the theory of Chosa et al. (2004), Terai et al. (2010), microstrain mechanical test sensor patches with a gauge length of 2 mm were placed on the middle of the L4 vertebral body isthmus and at the device attachment sites (spinous process root and lamina), respectively. Strain gauge adhesives were used to affix strain gauges to the corresponding bone surfaces. After the strain gauges were fixed, the sensor leads were connected to a static strain gauge (Boren Jizhi Technology Co., Ltd., Beijing, China) to collect real-time strain data under different conditions and were directly imported into a computer processing terminal (Figures 5A,B). Bone surface strain(ε) was measured experimentally. Based on prior studies (Dan et al., 2018), the surface Young’s modulus(E) of bovine calf bone was inferred to be 12 GPa; accordingly, the bone surface stress(σ) was computed as σ = Eε.

**FIGURE 6:**
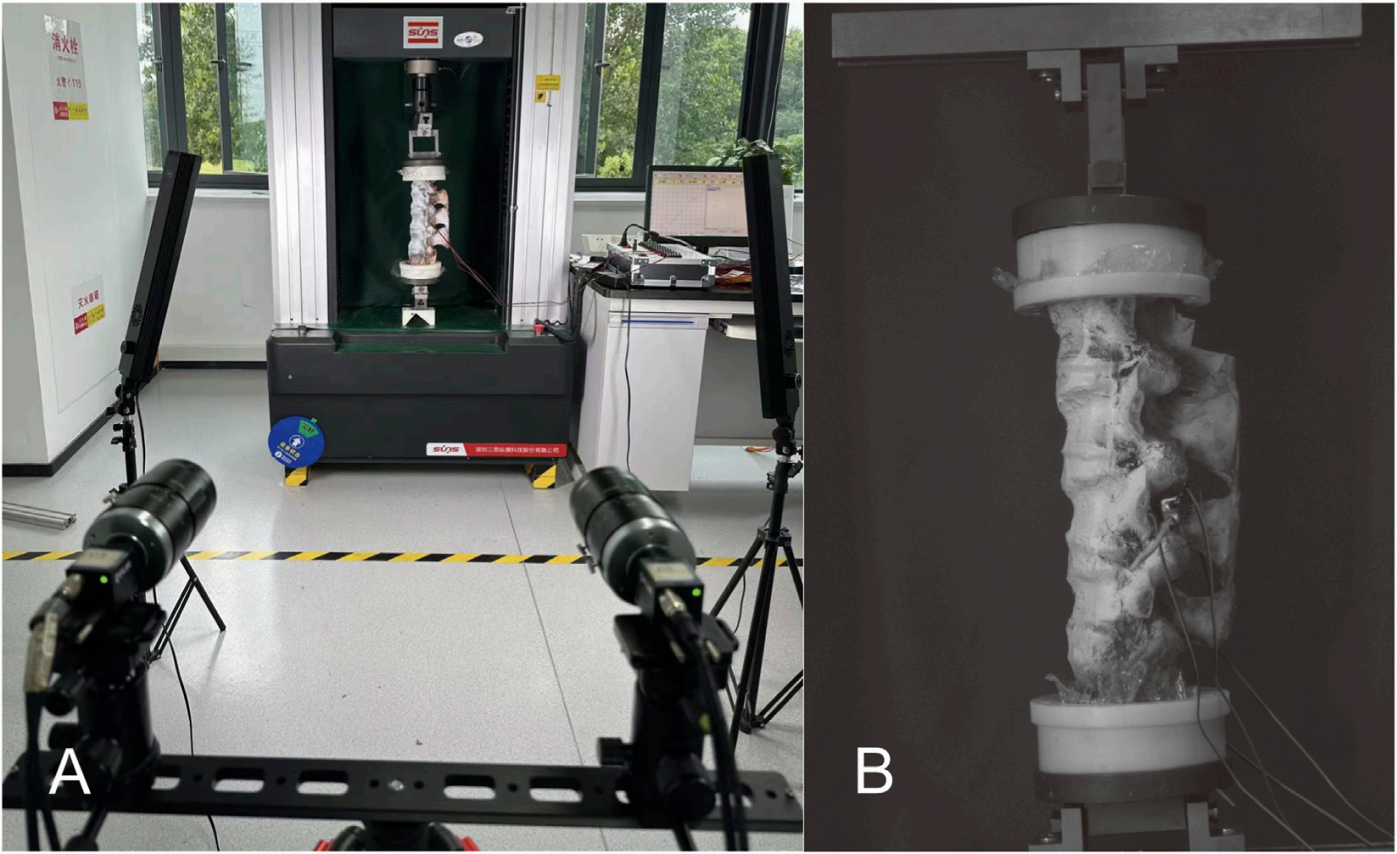
The method of measuring ROM. **(A)** Using optical strain measurement technology to dynamically measure the displacement of the surgical segment. **(B)** The projected image in the computer, the displacement angle of the fixed segment is calculated by the software.

A VIC-3D non-contact full-field measurement system (ACQTEC, Shanghai, China) was used to dynamically monitor the displacement changes in the surgical segment. The measurement accuracy of this system was 0.01 mm, and the sampling frequency was 5 Hz (Figures 6A,B). The experimental test sequences were: INT, IS, PSH, PSU, and PSDR. To ensure the reliability of the data, a repeated loading scheme was adopted, where each loading mode was tested three times, and the final measurement value was used for the analysis to eliminate errors caused by material viscoelasticity. Additionally, a 4-min recovery period was established after each surgery to allow the specimens to fully recover, thereby minimizing the impact of the surgical procedure on their mechanical properties (Luca et al., 2013).

### 2.4 Statistical analysis

Experimental measurements were grouped according to the direction of movement and expressed as mean ± standard deviation (mean ± SD) to compare the differences between different surgical methods. Statistical analysis was performed using one-way repeated-measures analysis of variance (ANOVA). If there were significant differences between groups, Tukey’s *post hoc* test was used for pairwise comparisons. Differences were considered statistically significant at P < 0.05. SPSS 28.0 (IBM, USA) was used for statistical analysis.

## 3 Results

### 3.1 Range of motion

**FIGURE 7:**
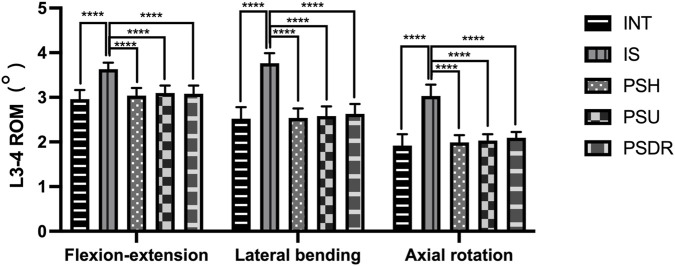
ROM of L3-4 with various fixation methods, statistically significant differences between the IS and others (****P < 0.0001). INT, intact lumbar; IS, isthmic spondylolysis; PSH, pedicle screw hook; PSU, pedicle screw U-shaped rod; PSDR, pedicle screw-rod-domino-rope.

**FIGURE 8:**
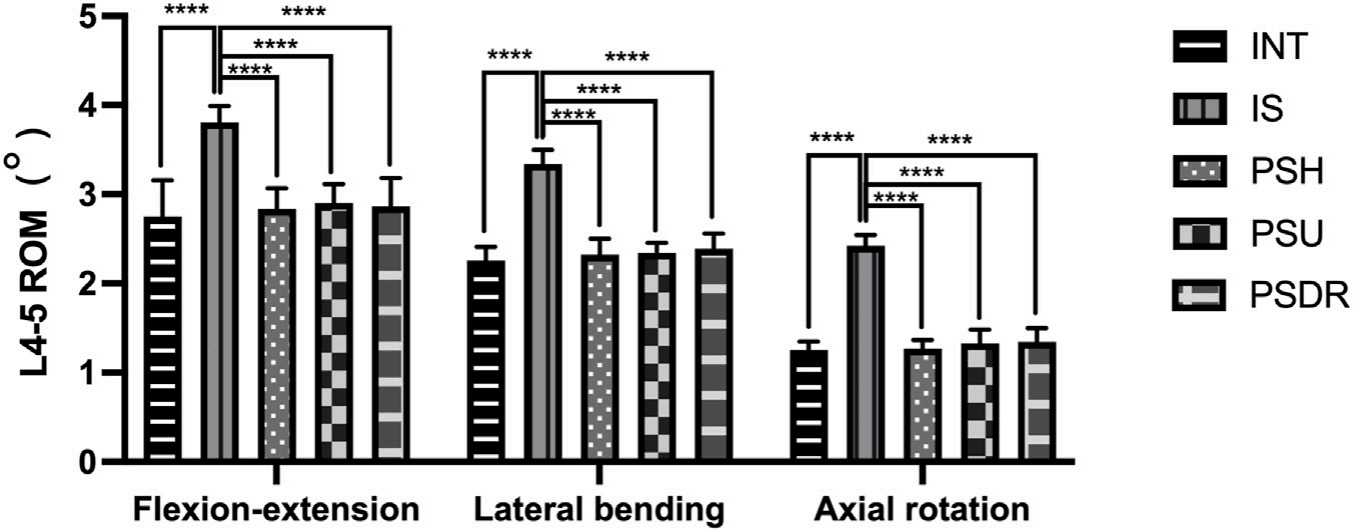
ROM of L4-5 with various fixation methods, statistically significant differences between the IS and others (****P < 0.0001). INT, intact lumbar; IS, isthmic spondylolysis; PSH, pedicle screw hook; PSU, pedicle screw U-shaped rod; PSDR, pedicle screw-rod-domino-rope.

Including the range of motion (ROM) of L3-4 (Table 1; Figure 7) and L4-5 (Table 2; Figure 8) segments.

**TABLE 1:** L3-4 ROM under different surgical models (°).

Motion	INT	IS	PSH	PSU	PSDR
Flexion-extension	2.96 ± 0.21	3.63 ± 0.15	3.04 ± 0.17	3.1 ± 0.17	3.08 ± 0.19
Lateral bending	2.52 ± 0.26	3.76 ± 0.22	2.54 ± 0.21	2.58 ± 0.22	2.63 ± 0.22
Axial rotation	1.92 ± 0.25	3.03 ± 0.26	1.99 ± 0.16	2.03 ± 0.14	2.1 ± 0.13

Values are presented as mean ± SD.

INT, intact lumbar; IS, isthmic spondylolysis; PSH, pedicle screw-hook; PSU, pedicle screw-U-shaped rod; PSDR, pedicle screw-rod-domino-rope.

**TABLE 2:** L4-5 ROM under different surgical models (°).

Motion	INT	IS	PSH	PSU	PSDR
Flexion-extension	2.75 ± 0.41	3.81 ± 0.18	2.84 ± 0.23	2.9 ± 0.21	2.87 ± 0.31
Lateral bending	2.26 ± 0.15	3.34 ± 0.16	2.32 ± 0.18	2.34 ± 0.11	2.39 ± 0.17
Axial rotation	1.25 ± 0.1	2.42 ± 0.12	1.27 ± 0.09	1.33 ± 0.16	1.35 ± 0.15

Values are presented as mean ± SD.

INT, intact lumbar; IS, isthmic spondylolysis; PSH, pedicle screw-hook; PSU, pedicle screw-U-shaped rod; PSDR, pedicle screw-rod-domino-rope.

#### 3.1.1 Flexion and extension

In both the L3-4 and L4-5 segments, the ROM of PSH, PSU, and PSDR was significantly lower than that of IS (P < 0.05). The decrease in ROM for each device at the L3-4 segment was 16.25%, 14.60%, and 15.15%, respectively. At the L4-5 segment, the decreases were 25.46%, 23.88%, and 24.67%, respectively. Compared with INT, the ROM of PSH, PSU, and PSDR showed no statistical significance at both segments (P > 0.05). Similarly, the ROM of the three device groups also showed no statistical significance at both segments (P > 0.05). However, numerically, PSU had the largest ROM at both segments. At the L3-4 segment, the increases were 0.06° and 0.02° compared to PSH and PSDR, respectively; at the L4-5 segment, the corresponding increases were 0.06° and 0.03°.

#### 3.1.2 Lateral bending

In both the L3-4 and L4-5 segments, the ROM of PSH, PSU, and PSDR was significantly lower than that of IS (P < 0.05). The reduction rates for each device at the L3-4 segment were 32.45%, 31.38%, and 30.05%, respectively. The reduction rates for each device at the L4-5 segment were 30.54%, 29.94%, and 28.44%, respectively. Compared with INT, the ROM of PSH, PSU, and PSDR was not statistically significant at both segments (P > 0.05). Similarly, the ROMs of the three device groups also exhibited no statistical significance at both segments (P > 0.05). Numerically, PSDR showed increases of 0.09° and 0.05° over PSH, and 0.07° and 0.05° over PSU at the L3-4 and L4-5 segments, respectively.

#### 3.1.3 Axial rotation

At both the L3-4 and L4-5 segments, the ROM of PSH, PSU, and PSDR was significantly lower than that of IS (P < 0.05). The decrease in ROM for each device at the L3-4 segment was 34.32%, 33.00%, and 30.69%, respectively. At the L4-5 segment, the decreases were 47.52%, 45.04%, and 44.21%, respectively. Compared to INT, there was no statistically significant difference in ROM between PSH, PSU, and PSDR at both segments (P > 0.05). Similarly, there was no statistically significant difference in ROM among the three device groups at both segments (P > 0.05). However, numerically, PSDR showed an increase compared to PSH and PSU at both segments. At the L3-4 segment, the increases were 0.11° and 0.07°, and at the L4-5 segment, they were 0.08° and 0.02°, respectively.

### 3.2 Stress values

The elastic modulus of the lumbar vertebral bone in calf specimens is 12 GPa (Dan et al., 2018), with a Poisson’s ratio of 0.30. In this study, 'device attachment site stress ' refers to the stress on the vertebral surface at the attachment point where the fixation instrument comes into contact with the bone.

#### 3.2.1 Lumbar spondylolysis stress

**FIGURE 9:**
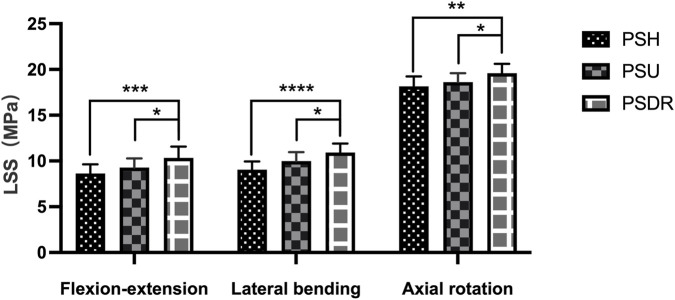
Lumbar spondylolysis stress with various fixation methods, PSDR differed significantly from PSH and PSU (*P < 0.05, **P < 0.01, ***P < 0.001, ****P < 0.0001). PSH, pedicle screw hook; PSU, pedicle screw U-shaped rod; PSDR, pedicle screw-rod-domino-rope.

Across the three activity states, significant differences were observed in the LSS, PSH, and PSU of the PSDR (P < 0.05). Specifically, PSU exhibited values that were 0.64 MPa and 0.46 MPa higher than those of PSH in the flexion-extension and rotation directions, respectively. The LSS of the PSDR was greater than that of both the PSH and PSU. Compared with PSDR, the LSS of PSH and PSU decreased by 16.54% and 10.35% in flexion-extension (P < 0.05), 17.22% and 8.52% in lateral bending (P < 0.05), and 7.35% and 5.00% in axial rotation (P < 0.05) (Table 3; Figure 9).

**TABLE 3:** LSS under different surgical models (MPa).

Motion	PSH	PSU	PSDR
Flexion-extension	8.63 ± 1.01	9.27 ± 1.01	10.34 ± 1.23
Lateral bending	9.04 ± 0.9	9.99 ± 0.97	10.92 ± 0.99
Axial rotation	18.16 ± 1.08	18.62 ± 0.97	19.6 ± 1.02

Values are presented as mean ± SD.

PSH, pedicle screw-hook; PSU, pedicle screw-U-shaped rod; PSDR, pedicle screw-rod-domino-rope.

#### 3.2.2 Device attachment site stress

**FIGURE 10:**
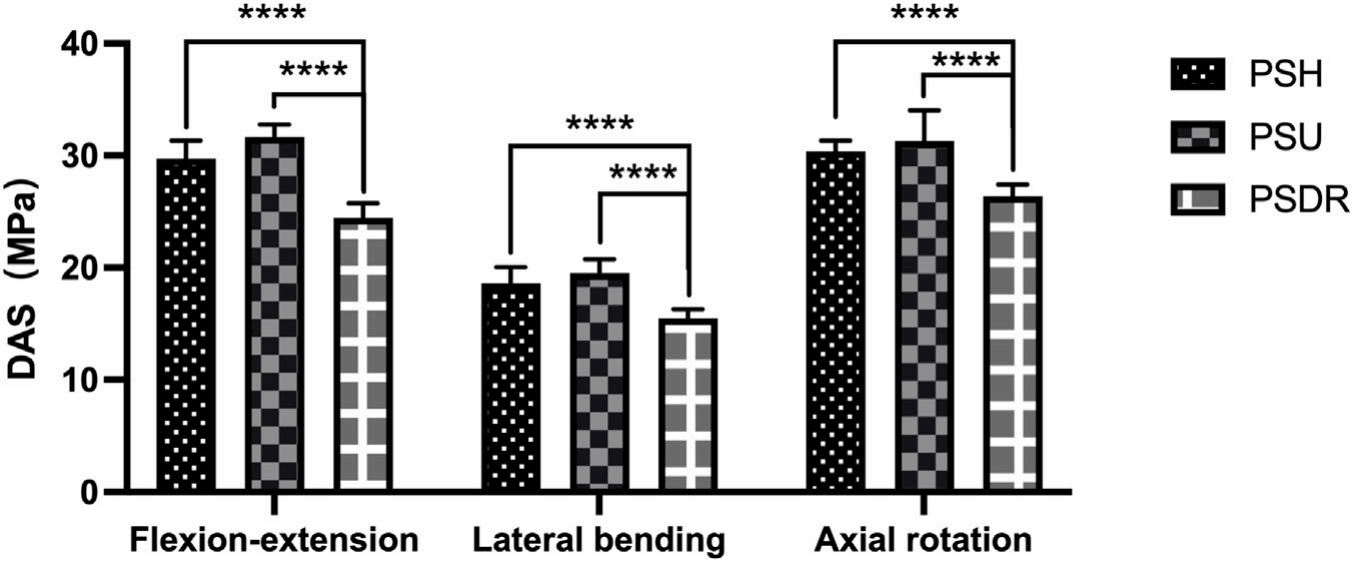
Device attachment site stress with various fixation methods: PSDR differed significantly from PSH and PSU (****P < 0.0001). INT, intact lumbar; IS, isthmic spondylolysis; PSH, pedicle screw hook; PSU, pedicle screw U-shaped rod; PSDR, pedicle screw-rod-domino-rope.

In the three activity states, the DAS of PSH and PSU were significantly greater than that of PSDR (P < 0.05), with increases of 21.42% and 29.35% in the flexion-extension direction, 20.14% and 25.95% in the lateral bending direction, and 15.2% and 21.34% in the rotational direction, respectively (Table 4; Figure 10).

**TABLE 4:** DAS under different surgical models (MPa).

Motion	PSH	PSU	PSDR
Flexion-extension	18.61 ± 1.44	19.51 ± 1.26	15.49 ± 0.79
Lateral bending	29.7 ± 1.62	31.64 ± 1.12	24.46 ± 1.3
Axial rotation	30.39 ± 0.93	32.01 ± 1.51	26.38 ± 1.04

Values are presented as mean ± SD.

PSH, pedicle screw-hook; PSU, pedicle screw-U-shaped rod; PSDR, pedicle screw-rod-domino-rope.

## 4 Discussion

When conservative treatment fails to alleviate symptoms in patients with symptomatic spondylolysis, surgical intervention may be considered (Tanveer et al., 2021). Most scholars advocate the use of intrasegmental fusion to repair spondylolysis directly (Li et al., 2022). Mohammed and other scholars (Mohammed et al., 2018) analyzed four types of segmental internal fixation techniques for spondylolysis. They found that the fixation method based on pedicle screws achieved the highest fusion rate and the lowest complication rate, making it the optimal choice for surgery. More representative repair methods include PSH (Tokuhashi and Matsuzaki, 1996) and PSU (Altaf et al., 2011). However, the PSH lamina hook needs to be placed close to the lamina, and improper placement or excessive compression may lead to lamina fractures. Patients with dysplastic or abnormal laminae may compromise the success rate and stability of the implantation. Furthermore, this technique causes extensive muscle damage, which may increase the operation time, blood loss, and risk of nerve injury (Guo et al., 2024; Zhang et al., 2023). PSU poses a risk of fracture due to stress concentration at the root of the spinous process, and intraoperative angle adjustment may prolong operation time and increase blood loss. Additionally, it may lead to separation of the fractured ends of the spondylolysis under flexion, thereby compromising the therapeutic outcome (Kim et al., 2024).

Nonfusion posterior dynamic stabilization devices have garnered attention owing to their ability to maintain stability in a semirigid environment and reduce the risk of implant fracture (Sun et al., 2025). Rohlmann et al. (2007) found through three-dimensional finite element analysis, that dynamic fixation in intrasegmental fusion of spondylolysis segments achieved similar stability to rigid fixation, and it also posed a lower risk of implant fracture. Zeng et al. (2017) used ISOBAR TTL dynamic fixation to treat lumbar spondylolysis, however, this technique may affect the lumbar range of motion and carry the risk of adjacent segment disease, thus requiring careful selection in adolescent patients. Berjano et al. (2020) employed pedicle screws, single rods, and polyester bands instead of steel cables, to repair lumbar spondylolysis. Dynamic fixation reduces the rigidity of internal fixation and the risk of steel cable fracture, and enhances the fusion rate of bone grafting. However, this technique required further clinical validation. On the basis of the aforementioned research, we designed a novel lumbar spondylolysis repair device. We previously verified the feasibility of PSDR through three-dimensional finite element analysis (Zhou et al., 2023). Building on this foundation, this study further conducted *in vitro* biomechanical experiments to compare the performance differences between PSDR and commonly used lumbar spondylolysis repair devices to explore its clinical performance.

The results indicated that compared to the intact state (INT), the ROM in isthmic spondylolysis (IS) significantly increased under three different motion states, leading to a decrease in lumbar stability. This may be the primary reason for the susceptibility to lumbar spondylolysis (Chung and Shimer, 2021). Following device implantation, PSH, PSU, and PSDR all significantly reduced lumbar ROM under the three different movement states, restoring them to levels similar to that of INT, indicating that all three devices effectively improved spinal stability.

Previous studies on device attachment site stress (DAS) and lumbar spondylolysis stress (LSS) in lumbar spondylolysis were based on three-dimensional finite element analysis, with few reports on *in vitro* biomechanical research. Our experimental results indicate that, compared to PSDR, the DAS of PSH and PSU increased by 21.42% and 29.35%, respectively, under flexion-extension conditions; by 20.14% and 25.95%, respectively, under lateral bending conditions; and by 15.2% and 21.34%, respectively, under rotational conditions. Among them, the increase in stress under flexion-extension conditions was the most significant, indicating that PSDR can effectively reduce the stress on the implant under these conditions. Previous studies have shown that dynamic fixation devices can significantly reduce the overall stress, thereby reducing the risk of screw loosening (Berjano et al., 2020; Michael et al., 2010; Pan et al., 2023; Rohlmann et al., 2007). Berjano (Berjano et al., 2020) found that rigid fixation devices are prone to fatigue fractures at the attachment site owing to long-term repeated stress stimulation, whereas dynamic fixation devices can effectively reduce such risks and avoid fixation failure caused by stress concentration. The PSDR adopts a dynamic fixation design, with both ends of the rope wrapped around the root of the spinous process, significantly reducing the DAS per unit area and avoiding the risk of excessive stress concentration.

In this experiment, PSDR exhibited the highest LSS. Compared to PSDR, the LSS of PSH and PSU decreased by 16.54% and 10.35% respectively in flexion-extension, 17.22% and 8.52% respectively in lateral bending, and 7.35% and 5% respectively in rotation. PSDR incorporates a PET rope device, providing a dynamic healing environment for the fractured ends of the spondylolysis. Compared to PSH and PSU, its average range of motion (ROM) increased by 0.07° and 0.02° respectively. Therefore, PSDR can provide maximum stress protection for spondylolysis and maintain a dynamic healing environment. According to Wolff’s law (Frost, 1994), the transition of the fixation device from a continuous stress mode resulting from rigid fixation to an intermittent dynamic stress mode of semirigid fixation can effectively promote spondylolysis healing. Barcik and Epari (2021) and Windolf et al. (2021) found that compared to continuous stress stimulation, fracture ends are more sensitive to periodic or intermittent stress stimulation.

Both PSDR and PSU apply stress to the root of the spinous process in structural design, inducing stress on the fractured end of the isthmus to promote healing. PSU may increase cephalad movement of the spinous process-lamina structure during flexion, thereby affecting the healing outcome (Kim et al., 2024; Li et al., 2022). Pan et al. (2023) designed a “W”-shaped fixation device that restricts movement by bypassing the top of the spinous process to promote healing; Kim et al. (2024) added a steel cable bypassing the top of the spinous process to the U-shaped rod on the basis of PSU, connecting it to counteract the anterior displacement of the spinous process-lamina, thereby improving the healing rate. However, both devices are rigid fixations, which may increase the risk of spinous process fractures owing to the stress concentration. In this study, PSDR utilized PET ropes and domino devices to restrict the movement of spinous processes in multiple directions. This approach not only mitigated the risk of excessive stress concentration on the spinous processes but also effectively prevented the separation of the isthmic fracture ends. The results indicated that, compared to PSU, PSDR reduced the range of motion (ROM) by an average of 0.025° in both flexion and extension states, suggesting its ability to limit spinous process-lamina displacement to a certain extent.

The PET rope utilized in this experiment possesses a low elastic modulus, high strength, flexibility, antimicrobial properties and good fatigue resistance, which can provide a stable dynamic healing environment for the defect site of lumbar spondylolysis fractures (Morais et al., 2020; Müller et al., 2001). It effectively withstands the load of the postoperative lumbar movement and reduces the risk of infection (Cipriani et al., 2013). Studies have shown that PET ropes causes minimal damage to the body and exhibit good biostability and biocompatibility, indicating that they can provide an ideal mechanical and biological environment for spondylolysis repair (Cipriani et al., 2013; Stoll et al., 2002).

The limitations of this study are as follows: 1) According to preoperative CT scans, the average bone density of the calf lumbar vertebrae was approximately 1.2 g/cm^3^, which is approximately 1.4 times higher than that of human bones, potentially affecting the experimental results. 2) This experiment only measured the strain at the fractured end of the isthmus and did not simulate the bone grafting and healing process of the isthmus, as well as the influence of muscle strength on isthmus healing; 3) The strain gauge was placed at the location of the maximum surface strain on the lumbar isthmus. It can only capture surface strain data and cannot accurately reflect mechanical changes within the isthmus. 4) Owing to the limited sample size, the statistical power of this study was low, posing a risk of false-negative results. Thus, further clinical studies are required to verify its long-term efficacy. 5) All five conditions were applied sequentially to the same specimen, sequence effects from cumulative manipulation cannot be fully excluded; however, a 4-min recovery, triplicate trials, and a fixed order were used to mitigate this risk. 6) The PSDR cables were preloaded with a force of 200N, but the optimal preload for this device remains to be further verified.

## 5 Conclusion

In this study, we assessed the biomechanical properties of a novel lumbar spondylolysis fixation device. The results showed that compared with PSH and PSU, PSDR could effectively control the ROM of the spine and restore nearly complete stability. However, PSDR could significantly reduce the stress at the device attachment site and increase the stress in the lumbar spondylolysis area. This dynamic fixation provides a new treatment approach for adolescent patients who have failed conservative treatment. However, further research using human cadaver specimens or clinical trials is necessary to evaluate the clinical efficacy of these devices in adolescent patients. While these metrics provide valuable insights into the biomechanical performance of different fixation devices, further studies are needed to evaluate fixation strength, cyclic fatigue, and clinical outcomes such as risk reduction for fractures or loosening.

## Data Availability

The original contributions presented in the study are included in the article/supplementary material, further inquiries can be directed to the corresponding authors.
